# Underwater Shockwave Pretreatment Process to Improve the Scent of Extracted* Citrus junos* Tanaka (Yuzu) Juice

**DOI:** 10.1155/2017/2375181

**Published:** 2017-07-06

**Authors:** Eisuke Kuraya, Akiko Touyama, Shina Nakada, Osamu Higa, Shigeru Itoh

**Affiliations:** National Institute of Technology, Okinawa College, 905 Henoko, Nago City, Okinawa 905-2192, Japan

## Abstract

*Citrus junos* Tanaka (yuzu) has a strong characteristic aroma and thus its juice is used in various Japanese foods. Herein, we evaluate the volatile compounds in yuzu juice to investigate whether underwater shockwave pretreatment affects its scent. A shockwave pretreatment at increased discharge and energy of 3.5 kV and 4.9 kJ, respectively, increased the content of aroma-active compounds. Moreover, the underwater shockwave pretreatment afforded an approximate tenfold increase in the scent intensity of yuzu juice cultivated in Rikuzentakata. The proposed treatment method exhibited reliable and good performance for the extraction of volatile and aroma-active compounds from the yuzu fruit. The broad applicability and high reliability of this technique for improving the scent of yuzu fruit juice were demonstrated, confirming its potential for application to a wide range of food extraction processes.

## 1. Introduction

Citrus fruits are widely cultivated in regions between tropical and temperate zones and include some of the most important commercial crops.* Citrus junos* Tanaka (yuzu), a sour fruit, is cultivated mainly in Japan and Korea. In Japan, its annual production amounted to approximately 22,900 tons in 2013. Yuzu produced in Rikuzentakata (Iwate prefecture) is known as the “Northern Limit Yuzu” (NLY) and is renowned for its pleasant aroma [1]. NLY is produced in small quantities and as a consequence, it tends to be expensive. In addition, Rikuzentakata sustained significant damage during the Great Tohoku Earthquake and Tsunami in 2011. Thus, NLY is currently being promoted as part of the reconstruction plan.

Almost all parts of the yuzu fruit, including its peel, juice, and seeds, are utilized. Compared to other citrus fruits, yuzu has a strong characteristic aroma and is well known for the pleasant fragrance of its outer rind. Therefore, yuzu is used industrially in the production of sweets, beverages, cosmetics, perfumes, and aromatherapy products [2, 3]. Important bioactive components present in yuzu fruits include vitamin C, *β*-carotene, flavonoids, limonoids, and dietary fiber. The predominant citrus fruit-derived flavonoids are glycosides that also function as antioxidants [4, 5]. Citrus limonoids such as limonin and nomilin are responsible for the bitter taste of citrus fruits and are characterized by their substituted furan moiety [5]. Owing to these characteristics, yuzu juice is commonly used in Japanese cooking. In the standard juicing process, high pressure is applied to the flavedo and/or albedo of the yuzu fruit, resulting in the extraction of ascorbic acid, flavanone glycosides, and odorants present in the juice. Owing to the high pressure employed, overextraction of bitter components (e.g., naringin, neohesperidin, and limonoids) [6] often occurs and for this reason, the pressure must be carefully controlled. As a consequence, it is difficult to obtain fruit juice with a strong aroma using the standard juicing processes.

Shockwaves propagate in plant media at rates exceeding the speed of sound, dividing into penetration and reflected waves upon a change in density. Underwater shockwaves cause instantaneous high pressure, splitting open cell structures and instantly generating multiple cracks on cell wall [7, 8]. The shockwave pretreatment liquefies vegetable material immediately (to a consistency similar to that of carrot juice) and simultaneously destroys cell structures. In previous studies, the effect of instantaneous high pressure produced by a conventional mixer blending extraction method contributed to increased yields in the tomato saponin, esculeoside A, from tomatoes [9] and an increased extraction efficiency of lipophilic gingerols and shogaols from ginger [10]. In a recent study, we have shown that multiple cracks generated by underwater shockwaves act as permeation pathways, increasing the extraction ratio of essential oils in steam distillation processes [7]. This observation indicates that the implementation of underwater shockwave treatment as a preprocessing step can be useful in the extraction of functional components from food materials.

Ultrasound-assisted extraction is also known to have a significant effect on the rates of various processes in chemical and food industries. Using ultrasound, full extractions can be completed in minutes with high reproducibility; moreover, solvent consumption is reduced and a final product with higher purity is yielded [11]. The effects of ultrasound propagation in solid/liquid media have been described in the literature previously [12, 13]. Pulsed electric field (PEF) processing is a nonthermal food processing technology based on the application of short pulses of high voltage to the food product. This process has been investigated for its suitability to enhance the extractability of fruit and vegetable juices as well as intracellular compounds [14]. In one such application, a higher concentration of bioactive compounds, for example, polyphenols in tomatoes, was reported after the PEF treatment [15]. Boussetta et al. investigated the effects of applying PEF and high voltage electrical discharges on the efficiency of the extraction of total soluble matter and polyphenols from grape skins (*Vitis vinifera* L.), and observed large differences in the total amount of polyphenols before and after the treatment [16, 17]. However, before the extraction of intracellular metabolites in vegetables and fruits can take place, these processing methods require liquefaction. This allows the rapid fragmentation of the raw material and generation of cavitation bubbles that increase the electrical conductivity and permeability of the whole plant tissue sample in solid/liquid media.

Following our shockwave pretreatment method, the cell walls disintegrated and the fruit softened, thus allowing access to the juice. This pressurization method also facilitated efficient extraction of functional compounds from the fruit tissue. We have recently reported that the multiple cracks generated by underwater shockwaves act as permeation pathways, thereby increasing the extraction ratio of essential oils in steam distillation processes [7]. Moreover, we observed that the flavanone glycoside content and oxygen radical absorbance capacity value of yuzu juice increased ca. 1.7-fold following the underwater shockwave pretreatment of the whole fruit with pilot-scale processing equipment [8]. These results indicate that efficient extraction of ascorbic acid, flavanone glycosides, and limonoids from yuzu fruit can be achieved using underwater shockwave treatment as a preprocessing step. Moreover, the dynamic control of instantaneous high pressure by underwater shockwaves proves its feasibility as a preprocessing step in the extraction of functional components from food materials.

In this study, we introduce an innovative application for this pretreatment process, aimed at improving the strength of scent extracted from* Citrus junos* Tanaka (yuzu) fruit juice. We also evaluate the content of volatile compounds in yuzu fruit juice to investigate whether such a pretreatment method affects its characteristics.

## 2. Material and Methods

### 2.1. Plant Material

Fruits of NLY produced in Rikuzentakata (Iwate prefecture) and yuzu produced in Kochi (KY; grown in Kami city, Kochi prefecture; Kumon variety) were provided by the Iwate Agricultural Research Center. All fruits were fully ripe and firm when harvested in November 2013. For both types of fruit, the juice was extracted by hand pressing using a hand-operated citrus juicer (Nanyo LLC., Tokushima, Japan). The samples of yuzu juice examined were comprised of mixtures of 30 and 12 fruit samples from KY and NLY, respectively. The juice samples were filtered through a nonwoven fabric net and the extract was immediately prepared for analysis.

### 2.2. Chemicals

*n*-Hexane, sodium sulfate, and 1-pentanol (internal standard, special grade reagent) were purchased from Nacalai Tesque, Inc. (Kyoto, Japan). Distilled and deionized water was used in all aqueous solutions.

### 2.3. Underwater Shockwave Pretreatment

Pilot-scale processing equipment was developed for the pretreatment of the whole yuzu fruit with continuous underwater shockwaves [8]. The high voltage supply generated a voltage of 2.5–3.5 kV and the underwater shockwaves were generated temporarily in the vessel by electrical discharge. A whole piece of yuzu fruit was placed in a silicone tube (ID = 114 mm, OD = 124 mm), separated from the shockwave generation source, and subjected to the instantaneous high-pressure load produced by the underwater shockwave. On the basis of a previous study [18], we assumed that the pressure produced by the shockwave generator at 3.5 kV and 4.9 kJ was ~40 MPa. The volatile compound content was evaluated to determine whether the pretreatment affects the characteristics of yuzu juice.

### 2.4. Isolation of Volatile Compounds Present in Yuzu Juice

The juice sample (1 mL) was extracted with distilled* n*-hexane (1 mL). After adding the extraction solvent (*n*-hexane), the juice sample was sonicated for 5 min and allowed to stand at 4°C for 24 h. The hexane layer was separated and dried over anhydrous sodium sulfate. Aromatic characterization of the volatile compounds was carried out by gas chromatography-mass spectrometry (GC/MS).

### 2.5. Analysis of Volatile Components in Extracted Juice

The juice aroma profiles were evaluated to determine whether the pretreatment affected the characteristics of yuzu juice. Two sampling methods were employed, namely, the* n*-hexane extraction method and the headspace method. The juice samples extracted with* n*-hexane were analyzed using a gas chromatograph coupled to a mass spectrometer (QP-2010 Plus; Shimadzu Co., Kyoto, Japan) and equipped with an autoinjector (AOC-20i, Shimadzu). Quantitative determinations of volatile components were based on the analyses of peak areas. Furthermore, the aroma characteristics of the yuzu juice sample (close to sensory evaluation) were analyzed directly by headspace (HS)-GC/MS. In qualitative analysis, 0.2% (v/v) 1-pentanol was used as the internal standard. The internal standard solution (100 *μ*L) was added to the yuzu juice sample (diluted 100-fold; 1.9 mL) in a 20-mL gas-tight vial sealed with a septum. Using a headspace sampler (TurboMatrix HS-40, PerkinElmer, Inc., MA, USA), the sample vial was pressurized above the capillary column head pressure with a carrier gas (helium) and heated at 60°C for 35 min to equilibrate with the vapor-phase extraction. The pressurized vapor phase, including the volatile compounds, was subsequently transferred to the GC/MS system with an injection time of 0.05 min.

GC/MS analyses were performed using a ZB-WAX Plus column (length = 60 m, ID = 0.32 mm, and thickness = 0.5 *μ*m; Phenomenex Inc., Torrance, CA, USA). The GC oven temperature program was set as follows: 40°C held for 1 min, increased at 10°C/min to 160°C, increased at 5°C/min to 200°C, and held for 4 min. The injector and detector temperatures were set at 200°C. The mass range was scanned from 30 to 600 amu. Control of the GC/MS system and data peak processing were performed using Shimadzu GC/MS solution software (Version 2.7). The volatile components were identified by comparing their retention indices and mass fragmentation patterns with MS libraries (NIST05 and FFNSC Library ver. 1.2; Shimadzu Co., Kyoto, Japan). The volatile components were identified based on their linear retention indices (RIs) and by comparison of their mass spectra with the MS data of reference compounds. The linear RIs were determined for all constituents using a homologous series of* n*-alkanes (C8–C24), injected under the same chromatographic conditions as the samples. The odor-activity of all yuzu oil compounds was evaluated based on their flavor dilution (FD) factor values obtained using GC-olfactometry and aroma extract dilution analysis (AEDA) by Lan-Phi et al. [3].

## 3. Results and Discussion

We employed an underwater shockwave treatment method as a preprocessing step for the extraction of volatile components from yuzu samples. By comparing the results obtained from the analyses of two different yuzu varieties, we elucidated successfully the effect of shockwave treatment on NLY and established how it compares to its effect on KY. The weight of the fruit, production district, and cultivar are listed in Table 1. The weights of KY and NLY fruits were calculated using the average of the weights recorded for 30 and 12 fruits, respectively. For both varieties, the juice samples consisted of a mixture of 30 (for KY) and 12 (for NLY) fruit samples. In general, Kochi is the largest yuzu-producing district owing to its mild climate. Although it is possible to grow yuzu in Rikuzentakata, the fruit is generally smaller as a result of the colder climate of the Tohoku district. The weights of the KY and NLY fruits were calculated to be 129.6 ± 8.7 and 81.3 ± 14.0 g/fruit, respectively. As predicted, the average weight of the NLY fruit was lower than that associated with the KY fruit.

In this study, pilot-scale processing equipment was developed for the pretreatment of yuzu fruits with underwater shockwaves. It was possible to continuously feed yuzu fruits into the underwater shockwave treatment vessel [8]. Following exposure of the whole fruit to the shockwave treatment, the oil glands were crushed and volatile oils and juice were released from the fruit. This was attributed to the spalling destruction caused by the shockwave treatment, whereby the resulting cracks could act as permeation pathways for the aroma compounds during the extraction process. These observations prove that the underwater shockwaves reached the entire cell and readily destroyed the fruit oil glands and cell walls.

The volatile compounds were analyzed by GC/MS and identified by their peak areas and comparison to mass spectrometry libraries. We identified 19 compounds in the* n*-hexane juice extract, including limonene, *γ*-terpinene, myrcene, *α*-pinene, *β*-pinene, *β*-phellandrene,* p*-cymene, and *α*-terpinolene (Table 2). Notably, the volatile contents in the extracted KY and NLY juice samples were distinctly different. The shockwave (SW) pretreatment afforded negligible changes in the relative concentrations of the two major volatile components in KY limonene (control = 86.8%; with pretreatment = 83.5–85.3%) and *γ*-terpinene (control = 7.19%; with pretreatment = 7.90–8.49). Negligible changes were also observed in the main volatile compounds of NLY extracts (limonene; control = 72.5%; with pretreatment = 71.4–71.7%). Interestingly, the relative concentrations of sabinene and caryophyllene were not detected in the untreated and SW 2.5 kV-treated NLY juice extracts but increased after a shockwave treatment of 3.5 kV was employed. These results indicate that the oil glands distributed in the yuzu peel are effectively destroyed by the SW treatment of 3.5 kV (but not 2.5 kV), and the multiple cracks created by the shockwave processing act as permeation pathways, increasing the extraction ratio of volatile compounds obtained during extraction.

**Figure 1:**
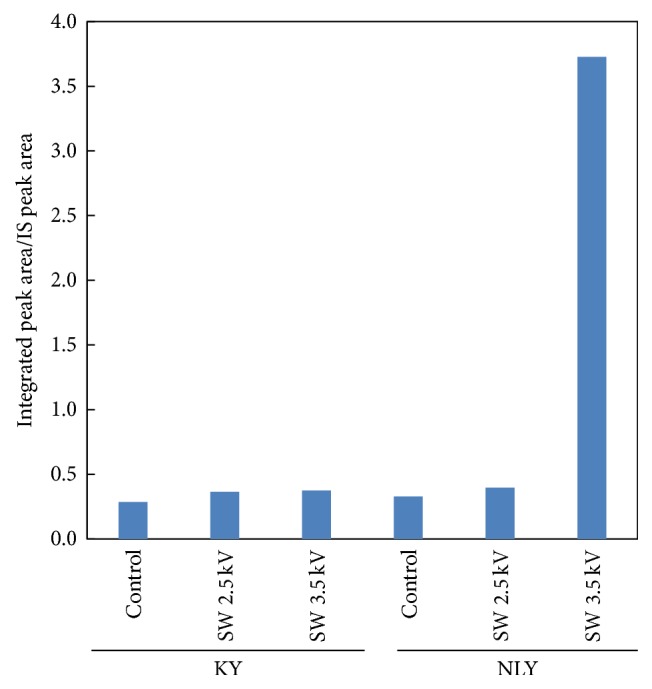
Changes in the content of volatile components in samples of yuzu juice as a function of treatment (SW represents shockwave treatment). The contents of volatile compounds were calculated from the ratio of the integrated areas of aroma components to the internal standard area.

The volatile content in treated and untreated yuzu fruits also differed, suggesting that, in addition to increasing the content of volatile compounds in the juice samples, the shockwave treatment also changed the scent intensity of the juice. The aroma characteristics of the citrus juice samples were analyzed by HS-GC/MS; this is close to sensory evaluation.  Figure 1 illustrates the contents of volatile compounds calculated from the ratio of the integrated area of the aroma component relative to the area arising from the internal standard. The contents of volatile components of untreated KY and NLY fruits were determined as 0.287 and 0.329, respectively, indicating that NLY fruit juice exhibits stronger scent intensity than KY juice. The volatile content of KY juice increased with an increase in applied shockwave energy (~×1.3 at 3.5 kV). However, for NLY, a more marked increase (maximum concentration ratio = 3.72; ~×11.3 increase) was observed when SW 3.5 kV was applied.

The odor-active components in yuzu oils, determined on the basis of the flavor dilution (FD) factor value, are important in the evaluation of the aroma characteristics of juice samples. The dilution of odor-active compound was performed until no odor was detected in the most diluted sample. The highest dilution at which an individual component could be detected was defined as the FD factor for that odorant [3, 19]. Thus, volatile compounds with high FD values contribute to the yuzu flavor. These compounds include limonene, *β*-phellandrene, and *γ*-terpinene (major volatile compounds) together with *α*-pinene, myrcene,* p*-cymene, *α*-terpinolene, linalool, (E)-*β*-farnesene, and bicyclogermacrene (trace volatile compounds). In this study, the contents of these components were evaluated to determine whether the shockwave pretreatment affects the characteristics of yuzu juice. The contents of major volatile components of the examined juice samples are listed in Table 3. Limonene, *β*-phellandrene, and *γ*-terpinene represent the odor-active components associated with yuzu juices and are described as having citrus-like aroma characteristic to the Kumon cultivar. Employing shockwave pretreatment at SW 3.5 kV increased the content of all of these components and thus, the odor-active content ratio of NLY fruit increased substantially (≥ ×10).

The contents of trace volatile components of juice samples are listed in Table 4. Although the contents of these compounds were low, their FD values were high, thus contributing strongly to the aroma of yuzu juice. Employing shockwave pretreatment at 3.5 kV increased the contents of all of these components. Notably, the odor-active content ratio of NLY increased substantially and the content of each compound was ca. ×10 greater than the increase observed for the major components. These results indicate that shockwave processing leads to the destruction of fruit oil glands and formation of multiple cracks, thus resulting in improved passage of volatile components from the yuzu fruit and more efficient extraction of volatile compounds, as well as a substantial increase in the odor of yuzu juice.

## 4. Conclusion

This study demonstrates the applicability and reliability of the underwater shockwave preprocessing treatment to improving the characteristics of odorants extracted from yuzu juice. This technique is reliable and exhibits excellent results in the extraction of volatile and aroma components from yuzu fruit prior to the extraction process. This underwater shockwave pretreatment technique can be also applied to other plant materials through the same mechanism, even if the odorants may be stored in different parts of the biological material. Moreover, this innovative process has the potential to find application in a wide range of extraction processes such as juice extraction or extraction of components from other naturally occurring foods and medicinal plants. Thus, the dynamic control of instantaneous high pressure by underwater shockwaves should prove valuable in many industrial applications.

## Figures and Tables

**Table 1:** Production districts, cultivars, and average weights of tested yuzu fruits.

Sample	Producing district	Cultivar	Weight of fruit (g)
KY^a^	Kami City, Kochi pref.	Kumon	129.6 ± 8.7
NLY^b^	Rikuzentakata, Iwate pref.	No name	81.3 ± 14.0

^a^ *n* = 30; ^b^*n* = 12.

**Table 2:** Volatile composition of yuzu juices.

Compound	RI	Relative concentration (%)					
		KY			NLY		
	ZB-WAX Plus	Control	SW 2.5 kV	SW 3.5 kV	Control	SW 2.5 kV	SW 3.5 kV
*α*-Pinene	990	1.51	1.52	1.72	1.81	1.89	2.15
*α*-Thujene	993	—	—	—	0.08	0.16	0.11
*β*-Pinene	1077	0.43	0.48	0.50	1.11	1.13	1.15
Sabinene	1090	—	—	—	—	—	0.12
Myrcene	1127	2.44	2.23	2.20	1.86	1.91	1.94
*α*-Phellandrene	1135	0.10	0.33	0.17	0.75	0.79	0.78
*α*-Terpinene	1149	0.17	0.16	0.15	0.32	0.29	0.32
Limonene	1169	86.77	83.50	85.26	72.46	71.42	71.70
*β*-Phellandrene	1180	0.40	1.59	0.91	4.06	4.55	4.43
*γ*-Terpinene	1215	7.19	8.49	7.90	14.65	14.85	15.14
*p*-Cymene	1242	0.32	0.42	0.42	0.80	0.97	0.78
*α*-Terpinolene	1253	0.29	0.27	0.26	0.44	0.43	0.42
Copaene	1462	0.01	0.04	0.02	0.01	0.03	0.02
Linalool	1495	0.06	0.46	0.28	1.06	0.87	0.35
Caryophyllene	1573	—	—	—	—	—	0.07
(E)-*β*-Farnesene	1613	0.04	0.22	0.08	0.30	0.25	0.30
*γ*-Cadinene	1682	0.16	0.11	0.08	0.07	0.09	0.05
Bicyclogermacrene	1706	—	—	—	0.07	0.06	0.07
*δ*-Cadinene	1721	0.02	0.06	0.04	0.03	0.06	0.03

Total		99.89	99.88	99.97	99.89	99.77	99.92

RI: Retention Index values were determined relative to *n*-alkanes on the ZB-WAX Plus column. The correct isomer was not identified. SW represents shockwave treatment.

**Table 3:** Overview of major volatile compounds in yuzu juice.

Compound	FD Value^a^	Intensity ratio of volatile component to internal standard					
		KY			NLY		
	log_3_ (FD-factor)^b^	Control	SW^c^ 2.5 kV	SW^c^ 3.5 kV	Control	SW^c^ 2.5 kV	SW^c^ 3.5 kV
Limonene	9	0.25	0.30	0.32	0.24	0.28	2.67
*β*-Phellandrene	8	0.001	0.006	0.003	0.013	0.018	0.165
*γ*-Terpinene	6	0.020	0.031	0.030	0.048	0.059	0.565

^a^Reference: Lan-Phi et al., 2009 [3].   ^b^The base-3 logarithm of flavor dilution factor value on DB-WAX column. ^c^SW represents shockwave treatment.

**Table 4:** Overview of trace volatile compounds in yuzu juice.

Compound	FD Value^a^	Intensity ratio of volatile component to internal standard (×10^−3^)					
		KY			NLY		
	log_3_ (FD-factor)^b^	Control	SW^c^ 2.5 kV	SW^c^ 3.5 kV	Control	SW^c^ 2.5 kV	SW^c^ 3.5 kV
*α*-Pinene	8	4.32	5.56	6.43	5.94	7.51	80.1
Myrcene	8	7.00	8.15	8.27	6.13	7.59	72.0
*p*-Cymene	8	0.89	1.52	1.56	2.63	3.84	29.3
*α*-Terpinolene	9	0.81	1.00	0.96	1.44	1.72	15.7
Linalool	7	0.18	1.66	1.04	3.48	3.44	12.8
(E)-*β*-Farnesene	9	0.11	0.81	0.31	1.00	1.00	11.1
Bicyclogermacrene	8	—	—	—	0.22	0.26	2.81

^a^Reference: Lan-Phi et al., 2009 [3].   ^b^The base-3 logarithm of flavor dilution factor value on DB-WAX column. ^c^SW represents shockwave treatment.
